# A Five-Gene Signature for the Prediction of Event-Free Survival of Both Pediatric and Adult Acute Myeloid Leukemia

**DOI:** 10.3390/diagnostics15111421

**Published:** 2025-06-03

**Authors:** Dechang Chen, Alvin J. Liu, Li Sheng, Zhenqiu Liu, Irina Elcheva

**Affiliations:** 1 Department of Preventive Medicine and Biostatistics, Uniformed Services University of the Health Sciences, Bethesda, MD 20814, USA; dechang.chen@usuhs.edu; 2 Department of Cognitive Science, Case Western Reserve University, Cleveland, OH 44106, USA; alvin.j.liu23@gmail.com; 3 Department of Mathematics, Drexel University, Philadelphia, PA 19104, USA; lsheng@math.drexel.edu; 4 Department of Statistics, Radiation Effects Research Foundation, Hiroshima 732-0815, Japan; 5 Division of Pediatric Hematology and Oncology, Department of Pediatrics, Penn State College of Medicine, 500 University Drive, Hershey, PA 17033, USA

**Keywords:** gene signature, event-free survival, adult and pediatric AML, expression profiles

## Abstract

**Background:** While adult and pediatric acute myeloid leukemia (AML) exhibit genetic distinctions, investigating their common gene expression program is critical for understanding the fundamental biological mechanisms that drive diverse cellular responses. However, existing gene signatures, predominantly tailored for overall survival (OS), may not adequately forecast event-free survival (EFS). EFS represents the time patients survive without disease recurrence, progression, or further treatment, a crucial metric for evaluating drug efficacy and assessing the clinical benefits of treatment. **Methods:** We performed an integrated analysis of adult TCGA and pediatric TARGET expression datasets to pinpoint genes and pathways associated with EFS in both adult and pediatric AML. Additionally, we constructed a predictive model using one dataset and validated it on the other to unveil a novel gene signature. **Results:** A five-gene signature comprising F2RL3, IL2RA, MYH15, SIX3, and SOBP was identified for EFS for both adult and pediatric AML. The test Area Under the ROC Curves (AUCs) for the 2-year and 5-year cutoffs of adult TCGA data were 0.851 (95% CI (Confidence Interval): 0.778–0.923) and 0.848 (95% CI: 0.729–0.968), respectively. Similarly, the test AUCs for the 2-year and 5-year cutoffs of Pediatric TARGET data were 0.725 (95% CI: 0.640–0.811) and 0.695 (95% CI: 0.59–0.80), respectively. When patients were stratified into three equal-sized prognostic subtypes based on the five-gene test risk scores, the P-values of tertile partitions for TARGET and TCGA data were 2.32e−6 and 5.12e−14, respectively, indicating superior performance compared to cytogenetic risk stratification within the same data (TARGET: P = 0.0019; TCGA: P = 0.0086). Despite being identified for EFS, the five-gene signature successfully stratified patients into distinct OS groups across two additional independent datasets. **Conclusions:** This five-gene signature demonstrates robust performance in both EFS and OS risk prediction and might be clinically significant.

## 1. Introduction

Acute myeloid leukemia (AML) is a hematological malignancy arising from chromosomal rearrangements, genes’ mutations, and other genetic and epigenetic abnormalities accumulated in blood progenitor cells [1]. While the incidence of AML increases with age, it can manifest at any stage of life affecting both children and adults. AML is recognized as a clinically heterogeneous disease characterized by the uncontrolled proliferation and accumulation of immature myeloid blood cells. Despite advancements in intensive chemotherapy and combination regimens that have demonstrated some efficacy in patient response [2], AML remains a challenging disease to treat. The 5-year overall survival rates for AML vary across age groups, equivalent to 60–75% in childhood AML, 35–40% in young adults under 60 years old, and a stark 5–15% in patients older than 60 years [3]. Accurate risk classification plays a pivotal role in determining the appropriate treatment strategies, including the selection of chemotherapy types, the timing of stem cell transplantation, and eligibility for participation in clinical trials [4]. Traditionally, AML has been categorized into three distinct groups based on gene mutations and cytogenetics [1]. However, this classification primarily focuses on overall survival stratification and may not always predict event (disease)-free survival (EFS) adequately. For instance, in a study of core binding factor AML (CBF-AML), including recurrent genetic translocations **t(8;21)(q22;q22)** involving RUNX1-RUNX1T1 and **inv(16)(p13q22)** involving CBFB-MYH11 [5], multivariate analyses of that study showed that KDM6A mutation was associated with OS (P < 0.001) but not with EFS (P = 0.095) in the RUNX1::RUNX1T1 cohort. Similarly, FLT3-ITD mutation was associated with OS (P = 0.024) but not with EFS (P = 0.07) in the CBFB::MYH11 cohort.

Childhood and adult AML have been demonstrated to exhibit biological and clinical distinctions [3,6,7,8,9]. Recent advancements in genomic profiling have revealed notable disparities in the genetic landscape of AML between pediatric and adult patients. Pediatric AML exhibits higher frequency of structural abnormalities; only 20% of pediatric AML cases have a normal karyotype and less cooperative genetic alterations, such as mutations in genes FLT3, NPM1, and CEBPA, often found in adult AML. Conversely, epigenetic abnormalities caused by mutations in DNMT3A and TET2 are more commonly found in adult AML cases. However, there are shared genetic mutations and genomic signatures between pediatric and adult AML [6]. Recent advancements in RNA sequencing technologies have led to the proposal of several transcriptional gene signatures common for adult and childhood AML [6,10,11,12,13,14]. However, these gene signatures are primarily designed for prognostic predictions related to OS and may not consistently perform well when applied to EFS. EFS is a key primary endpoint that quantifies the time patients remain alive without disease recurrence, progression, or the need for additional therapy [15]. While it does not always provide an earlier assessment, EFS is particularly useful for evaluating treatment efficacy when OS data require years to mature [16]. By focusing on events occurring during or shortly after the study treatment, EFS can provide a clearer picture of that treatment’s direct impact. Identifying gene signatures tailored for the stratification of AML patients into distinct EFS groups is imperative. In this pilot study, our objective is to pinpoint transcriptional gene signatures linked to EFS in both adult and childhood AML, utilizing the TCGA (The Cancer Genome Atlas) and TARGET (Therapeutically Applicable Research to Generate Effective Treatments) RNA-sequencing data. We aim to investigate shared genes in pediatric and adult AML that are correlated with EFS, ultimately constructing a risk score model. This model can predict 2-year and 5-year survival, enabling the stratification of patients into distinct prognostic groups. Investigating common gene expression programs is essential for uncovering the shared molecular mechanisms that drive disease onset and progression across age groups. Identifying overlapping transcriptional signatures can reveal core pathways essential for leukemogenesis. Such insights may help refine classification, improve risk stratification, and guide the development of universal therapeutic strategies.

## 2. Materials and Methods

### 2.1. Data Sources

- TARGET childhood AML data: We retrieved the TARGET AML RNA-seq data from the Genomic Data Commons (GDC) at portal.gdc.cancer.gov/, accessed on 20 November 2020. This dataset comprises 187 subjects and 21,047 genes with nonzero read counts. Additionally, clinical metadata, including EFS and censored status, are accessible. EFS represents the time patients survive without disease recurrence, progression, or further treatment [15]. This dataset was already processed and normalized to account for variations in the sequencing depth and library size, and it was log2 transformed, allowing for accurate comparison.
- TCGA adult AML data: The TCGA RNA-seq gene expression data were acquired from the Cancer Genomics Portal at https://www.cbioportal.org/, accessed on 16 February 2019. This dataset consists of 173 samples, encompassing 20,531 raw gene counts. Normalization was conducted through log2 transformation and quantile normalization. Similarly to the childhood AML data, EFS, censored status, and other pertinent clinical information were also included.
- Other validation data: For further validation, we incorporated two independent microarray datasets—GSE37642 [13] and GSE12417 [14], accessed on 8 December 2020. Both datasets represent adult AML, providing only OS information. GSE37642 encompasses 136 patients with 21,225 probes, while GSE12417 includes 163 patients with 21,225 probes. The microarray data were preprocessed with log2 transformation and quantile normalization.

### 2.2. Methods

Genes associated with EFS for TCGA and TARGET were independently identified using a univariate Cox regression. Subsequently, common genes present in both TCGA and TARGET datasets were subjected to score model construction using the glmnet 4.0-2 and survminer 0.4.8 packages in R [17,18]. The assessment of the proportional hazard assumption was conducted through scaled Schoenfeld residuals. Various other software tools were employed for specific tasks in the analysis. The VennDiagram 1.7.3 package in R facilitated the visualization of Venn diagrams, the ggplot2 3.4.0 function in R was utilized for generating a bar chart depicting KEGG pathways, and the machine learning toolbox in MATLAB R2020a was instrumental in drawing receiver operating characteristic (ROC) curves.

First, we performed univariate Cox regression separately on the TCGA and TARGET datasets to identify genes associated with EFS. For each dataset, we performed univariate Cox regression for all genes and identified 5483 EFS-associated genes in TARGET and 1944 in TCGA using a significance threshold of P < 0.05. Of these, 170 genes were common to both datasets, exhibiting P < 0.05 and consistent coefficient signs.

We then performed regularized multivariate Cox regression using glmnet 4.0-2 [17] with the 170 common genes for each dataset separately. The regularization parameter *λ* was determined using 5-fold cross-validation. The data were randomly divided into five roughly equal-sized subsets (folds). In each iteration, the model was trained on four folds (80% of the data), while the remaining fold (20%) was used for validation. This process was repeated five times, with a different fold used for validation in each iteration. The model’s performance was averaged across all five iterations.

The model’s performance was evaluated using Harrell’s Concordance Index (C-index) [19]. We identified 11 genes with the best C-index of 0.89 using a penalized parameter λ=0.281 for the TCGA dataset, and 9 genes with the best validation C-index of 0.82 using λ=0.736. Among these, 5 genes (F2RL3, IL2RA, MYH15, SIX3, and SOBP) were shared between the TCGA and TARGET datasets.

Since the coefficients estimated by glmnet 4.0-2 with an $L_{1}$ penalty tend to be biased toward zero, we refitted a standard Cox regression model using the 5 selected genes for both TCGA and TARGET. This allowed us to obtain the score models separately for each dataset. Finally, we used the score model derived from one dataset to evaluate its performance on the other dataset.

Each dataset serves as an independent test set for the model built from the other dataset. Survminer 0.4.8 software (https://github.com/kassambara/survminer, accessed on 12 December 2020) was used to generate Kaplan–Meier plots and perform log-rank tests using the test scores. Survminer 0.4.8 is an R package providing functions for survival analysis and visualization. The package includes functions for drawing Kaplan–Meier curves, conducting log-rank tests, and visualizing hazard ratios from Cox proportional hazards models.

## 3. Results

One hundred and seventy genes, common to both TCGA and TARGET datasets, are associated with EFS and enriched within biological pathways.

**Figure 1:**
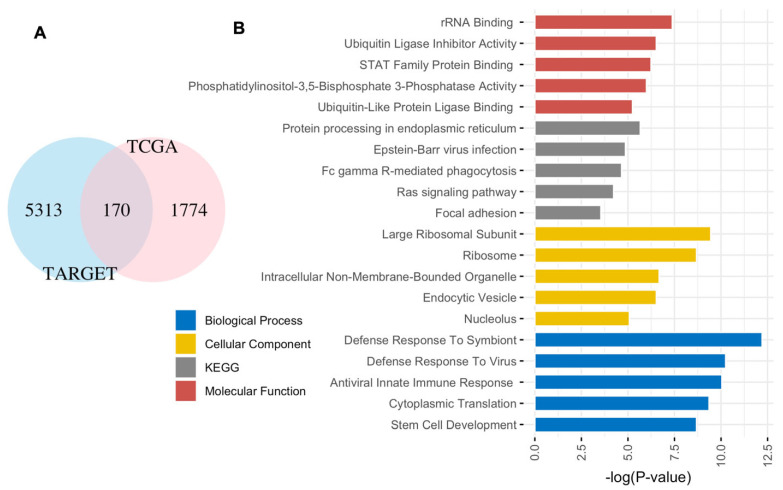
(**A**) Venn diagram illustrating the overlap of genes between adult and childhood AML. (**B**) Top 5 KEGG pathways and enriched biological functions.

We identified 170 genes shared between TCGA and TARGET datasets using the univariate Cox regression (P < 0.05), as illustrated in Figure 1 and detailed in Supplementary Table S1.

Figure 1A illustrates the identification of 170 genes associated with EFS shared between adult and pediatric AML. Supplementary Table S1 provides detailed information, including the 170 genes and their corresponding P-values in adult (TCGA) and pediatric AML. Moreover, a notable contrast emerges from the analysis of gene associations, revealing 5483 and 1944 genes linked to TARGET and TCGA, respectively. However, only 170 genes are shared between adult and pediatric AML, underscoring distinct gene expression and transcriptional patterns in these two cohorts.

Figure 1B presents the top 5 KEGG pathways, molecular functions, cellular components, and biological processes enriched with the 170 genes shared by both adults and pediatric AML. Molecular functions common to both include rRNA binding, ubiquitin ligase inhibitor activity, STAT family protein binding, phosphatidylinositol-3,5-bisphosphate 3-phosphatase activity, and ubiquitin-like protein ligase binding. Furthermore, the top five biological processes involve the defense response to the symbiont, defense response to the virus, antiviral innate immune response, cytoplasmic translation, and stem cell development. Cellular components associated with these genes include the large ribosomal subunit, ribosome, intracellular non-membrane-bounded organelle, endocytic vesicle, and nucleolus. The KEGG pathways implicated are protein processing in endoplasmic reticulum, Epstein–Barr virus infection, Fc gamma R-mediated phagocytosis, the Ras signaling pathway, and focal adhesion.

### 3.1. Five-Gene EFS Risk Score Models for Adult and Pediatric AML

Upon utilizing the glmnet R package [17] on the 170 genes shared between TCGA and TARGET, we formulated two EFS risk score models—one for adult (TCGA) and another for pediatric (TARGET) AML, respectively. The score derived from Cox regression represents the relative risk of an event occurring over time. The five-gene pediatric EFS risk score model, derived from TARGET data, is outlined as follows:

$$\begin{array}{c} Pediatric\;EFS\;Risk\;Score\;=\;0.1407\;\times \;F2RL3\;+\;0.3418\;\times \;IL2RA\;+\;0.2203\;\times \;MYH15\\ -0.2992\;\times \;SIX3\;+\;0.3473\;\times \;SOBP. \end{array}$$

And the five-gene adult EFS risk score model is as follows:

$$\begin{array}{c} Adult\;EFS\;Risk\;Score\;=\;0.7907\;\times \;F2RL3\;+\;0.6666\;\times \;IL2RA\;+\;1.6934\;\times \;MYH15\\ -0.3313\;\times \;SIX3\;+\;1.1803\;\times \;SOBP. \end{array}$$

Positive coefficients in the model are associated with poor EFS, while negative coefficients correlate with better EFS. The model incorporates the following five genes: F2RL3, IL2RA, MYH15, SIX3, and SOBP. Upregulation of SIX3 is linked to improved EFS, whereas upregulation of F2RL3, IL2RA, MYH15, and SOBP is associated with worse survival outcomes. Pediatric and adult risk scores can be readily calculated. For example, if the expression values for the five genes are all 1, the resulting pediatric and adult risk scores are 0.7509 and 3.9997, respectively.

**Figure 2:**
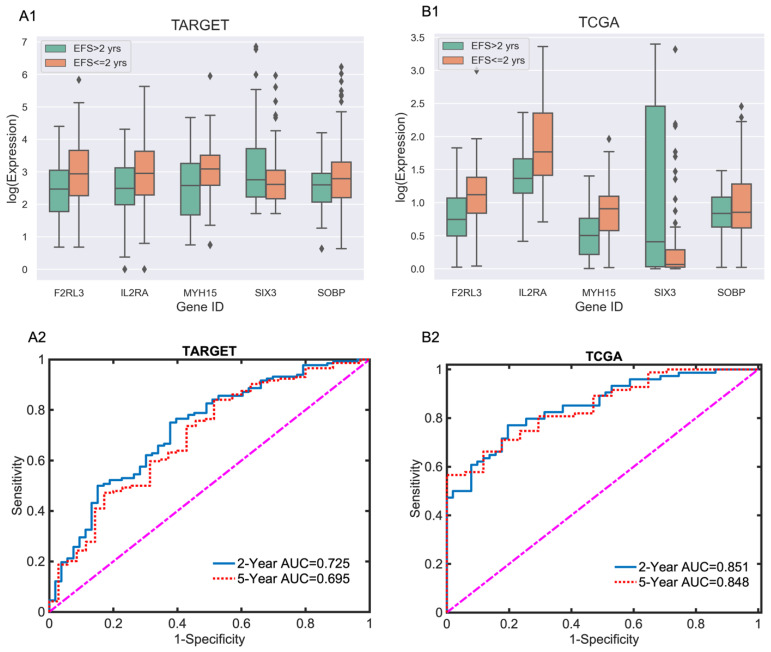
(**A1,B1**): Boxplots of the log expression values of the five marker genes for TARGET (**A1**) and TCGA (**B1**), stratified by EFS time ≤ 2 years and >2 years. (**A2**): Test AUCs for the 2-year (blue solid line) and 5-year (red dotted line) cutoffs in the (TARGET) pediatric data predicted by the adult EFS risk score model. (**B2**): Test AUCs for the 2-year and 5-year cutoffs in TCGA adult data, assessed using the pediatric EFS risk score model.

Figure 2 presents the log expression of five marker genes using a 2-year cutoff and the AUC performance of score functions at the 2-year and 5-year cutoff points.

As shown in Figure 2A1,B1, four marker genes—F2RL3, IL2RA, MYH15, and SOBP—exhibit higher expression in patients with an EFS time of less than 2 years, suggesting an association with poor EFS in both pediatric and adult AML. In contrast, SIX6 shows higher expression in patients with an EFS time of more than 2 years, indicating a potential link to better EFS.

We build the models using one set of data and assess their performance using the other set. Model performance is evaluated using the area under the receiver operating characteristic curves (AUCs). The test AUCs for the 2-year and 5-year cutoffs of EFS in adult (TCGA) data are assessed using the pediatric EFS risk score model. Conversely, the test AUCs for the 2-year and 5-year cutoffs of EFS in pediatric (TARGET) data are estimated using the adult EFS risk score model. The results of the test AUCs are presented in Figure 2A2 and 2B2, respectively.

Figure 2A2 illustrates that the test Area Under the ROC Curves (AUCs) for the 2-year and 5-year EFS cutoffs in the TARGET data are 0.725 (95% CI: 0.640–0.811) and 0.695 (95% CI: 0.59 –0.80), respectively. Meanwhile, the test AUCs for the 2-year and 5-year EFS cutoffs in TCGA (Figure 2B2) are 0.851 (95% CI: 0.778–0.923) and 0.848 (95% CI: 0.729–0.968), respectively.

### 3.2. The Five-Gene Signature Demonstrates Superior EFS Stratification Compared to Cytogenetics Method

**Figure 3:**
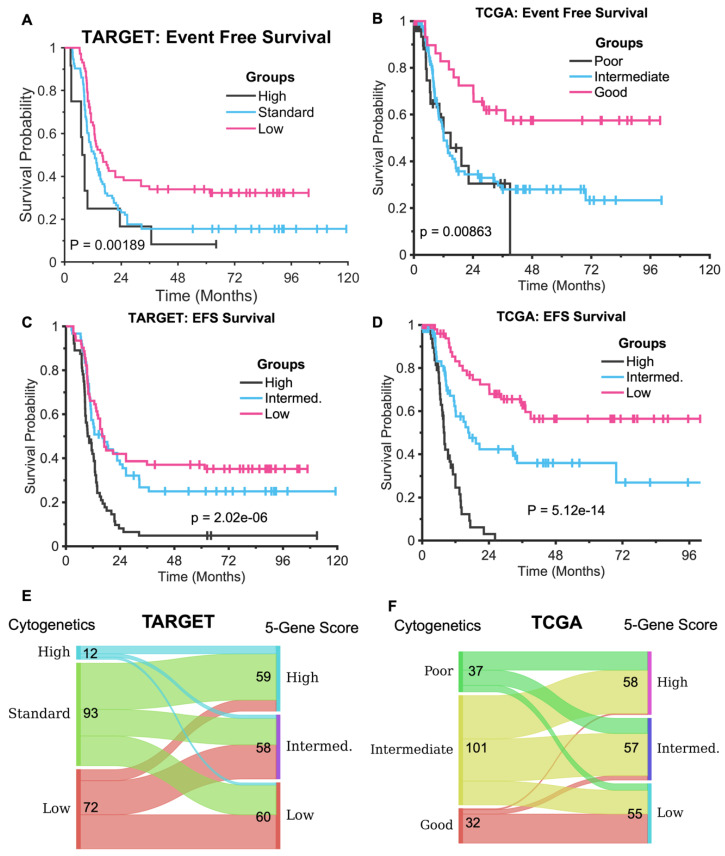
Kaplan–Meier curves for the TARGET and TCGA data. Top panel: Risk groups stratified by cytogenetics (**A**,**B**). Middle panel: (**C**): Risk groups of the TARGET data stratified by the adult (TCGA) risk score model. (**D**): Risk subtypes of TCGA grouped by the pediatric (TARGET) risk score model. (**E**,**F**): River (Sankey) plots demonstrate the group alignment of the patients between cytogenetic classification and subtypes based on the 5-gene score for TARGET (**E**) and TCGA (**F**), respectively. Patients with missing cytogenetic class information are excluded. The numbers beside the class names indicate the number of patients in the subtype.

Patient stratification into different risk groups is crucial for personalized treatments in AML. In AML, standard risk stratification based on cytogenetics plays a fundamental role in determining prognosis and guiding treatment decisions. Cytogenetics refers to the study of chromosomal abnormalities, which are common in AML and influence both the disease’s aggressiveness and response to therapy. Risk stratification typically categorizes patients into three main risk groups—low, intermediate, and high—based on specific chromosomal translocations, inversions, or deletions, as well as other genetic mutations. Recently, the three AML risk groups have been refined by integrating cytogenetic profiles with molecular mutations in several additional genes [20]. However, current risk stratification models are primarily defined based on overall survival, which may not accurately predict EFS. To further assess the efficacy of five-gene signatures in EFS risk stratification, we divided the TARGET and TCGA cohorts into three equal-sized subgroups using the other EFS risk score model. Subsequently, we compared their performance against the standard subtypes of cytogenetics, as depicted in Figure 3.

As depicted in Figure 3, Figure 3A,C present the Kaplan–Meier curves for the TARGET data, illustrating the standard cytogenetics and adult EFS risk score, respectively. Similarly, Figure 3B,D depicts the Kaplan–Meier curves for the TCGA data, showcasing their cytogenetics and pediatric EFS risk score. Compared to cytogenetics stratification, the risk groups identified by the five-gene EFS risk scores exhibited enhanced performance. The adult EFS risk score stratified TARGET patients into three roughly equal-sized groups with a P-value of 2.32e−06 and median EFS of 9.56, 12.88, and 26.58 months, respectively (Figure 3C). In contrast, the risk groups defined by cytogenetics had a larger P-value of 0.00189 and median EFS of 7.5, 12.9, and 16.0 months, respectively (Figure 3A). Importantly, the five-gene signature identified more patients (59) in the high-risk group, while only 12 patients were assigned to the high-risk group using cytogenetics. The cytogenetics classification failed to distinguish high EFS risk from intermediate EFS risk for pediatric AML. Figure 3E shows that only 12 patients are classified as high-risk based on the cytogenetic classification of the TARGET data. There is little consistency between cytogenetic classification and five-gene score stratification. A large proportion of patients classified as standard risk by cytogenetic classification are assigned to the high-risk subtype by the five-gene score. The discrepancy between risk score classification and cytogenetic classification likely arises from the fact that our five-gene signature captures molecular features beyond the known cytogenetic drivers. While cytogenetic classification is based on structural genomic alterations, our model leverages gene expression patterns that may reflect broader biological processes, including epigenetic regulation, transcriptional dynamics, and microenvironmental influences. Further investigation is needed to delineate how they contribute to risk stratification independently of cytogenetic markers. This is an important direction for our future research.

In the TCGA dataset, dividing patients into three equal-sized groups based on pediatric EFS risk scores (Figure 3D) demonstrated the superior performance of the five-gene signature, with a P-value of 5.12e−14 and median EFS of 7.8, 17, and NaN (unmeasurable based on the available data) months for the high-, intermediate-, and low-risk groups, respectively. In contrast, cytogenetic stratification yielded a P-value of 0.0086, with median EFS of 14.9, 12.1, and NaN (unmeasurable based on the available data) months for the poor-, intermediate-, and good-risk groups, respectively. Notably, the cytogenetic classification failed to distinguish between the poor- and intermediate-risk groups in terms of EFS, with the poor-risk group (median EFS of 14.9 months) showing a longer survival than the intermediate-risk group (median EFS of 12.1 months).

The five-gene signature effectively identified a high-risk group comprising 64 patients with a median survival time of only 7.8 months. Figure 3F shows that most patients classified as good-risk by cytogenetic classification are also assigned to the low-risk group. As a result, both the good-risk group in Figure 3B and the low-risk group in Figure 3D are well separated from other risk groups. However, there is little consistency in the other two risk groups when comparing cytogenetic classification and the five-gene score. Thus, no statistically significant EFS differences between the poor- and intermediate-risk groups are detected by cytogenetic classification (Figure 3B).

### 3.3. The Five-Gene Signature Predicts the Overall Survival of TARGET and TCGA and Two Independent Data

**Figure 4:**
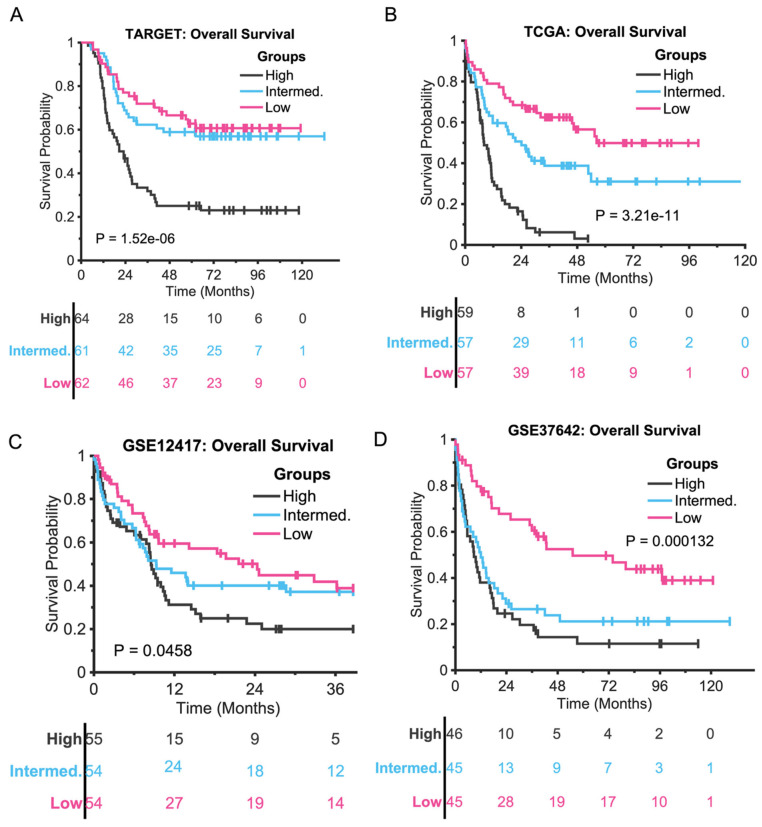
(**A**): Kaplan–Meier curves for TARGET, grouped by the coefficients of the adult risk score without re-estimating any parameters. (**B**–**D**): Kaplan–Meier curves for TCGA, GSE12417, and GSE37642, respectively, stratified by the coefficients of the pediatric EFS risk score.

Despite being identified for EFS, the five-gene signature demonstrates efficacy in predicting OS across various datasets, including two independent ones, as illustrated in Figure 4.

As illustrated in Figure 4, despite being developed for EFS classification, the five-gene signature effectively stratifies OS in the TARGET, TCGA, and two independent datasets (GSE12417 and GSE37642). The pediatric EFS risk score model divides the OS of TCGA data into three approximately equalized groups (Figure 4B) with a P-value of 3.21e−11 and median OS times of 8.1, 24.1, and 56.3 months for the high-, intermediate-, and low-risk groups, respectively. It also categorizes the OS time of GSE12417 into three roughly balanced groups (Figure 4C) with a P-value of 0.0458 and median OS times of 8.54, 9.30, and 24.35 months, respectively. Additionally, it divides the OS of GSE37642 into three roughly equalized subsets (Figure 4D) with a P-value of 0.000132 and median OS times of 8.71, 11.96, and 55.11 months, respectively. Furthermore, the adult EFS risk score classifies the OS time of TARGET data into three approximately equalized groups (Figure 4A) with a P-value of 1.52e−6 and median OS times of 23.06, NaN, and NaN months, respectively.

## 4. Discussion

Despite differences in the genetic mutations and clinical presentation, the 170 common genes and associated pathways indicate that fundamental biological mechanisms underlie both pediatric and adult AML. Certain core pathways drive AML pathogenesis regardless of patient age. For example, rRNA binding plays a crucial role in ribosome biogenesis and protein synthesis. Dysregulated ribosomal function has been linked to altered cell proliferation and survival, while increased ribosomal activity may contribute to the rapid growth of leukemic cells [21]. Similarly, ubiquitin ligase inhibitor activity disrupts the normal degradation of proteins involved in cell cycle regulation and apoptosis, stabilizing oncogenic proteins that drive AML. Targeting ubiquitin ligases has been explored as a potential therapeutic strategy [22]. Overall, the shared molecular functions, biological processes, cellular components, and pathways in pediatric and adult AML suggest that core oncogenic mechanisms remain consistent across age groups. These mechanisms include dysregulated protein synthesis, immune evasion, aberrant signaling pathway activation, and altered cellular interactions, all of which present potential therapeutic targets for AML treatment.

We identified a five-gene signature (F2RL3, IL2RA, MYH15, SIX3, and SOBP) that predicts 2-year and 5-year survival and stratifies patients into different EFS risk groups in both adult and pediatric tumors. Distinguishing a high-risk group in AML is crucial for improving treatment outcomes and personalizing therapeutic strategies. Patients in high-risk subgroup may require more intensive therapies, including higher-dose chemotherapy or hematopoietic stem cell transplantation. Conversely, lower-risk patients might benefit from less aggressive treatments, reducing exposure to toxicity without compromising efficacy. The five genes in the signature are clinically and biologically significant, with some being well-studied and others less extensively investigated.

The F2RL3 (coagulation factor II thrombin receptor-like 3), also known as PAR4, encodes a thrombin receptor involved in blood coagulation, inflammation, and cell signaling. Thrombin activation of F2RL3 may contribute to an inflammatory environment that supports AML cell survival, proliferation, and resistance to apoptosis. Additionally, F2RL3 might promote the maintenance of leukemic stem cells, which drive relapse and treatment resistance [23,24]. Our findings link upregulated F2RL3 with worse EFS in both adult and pediatric AML, highlighting its potential as a therapeutic target.

The IL2RA gene encodes the alpha chain of the IL-2 receptor, a key regulator of immune responses. AML is characterized by immune evasion, and IL2RA plays a role in T-cell signaling crucial for immune surveillance. Clinically, IL2RA serves as a biomarker for disease progression and a potential therapeutic target to disrupt the immunosuppressive AML environment [25]. Recent studies have identified IL2RA in risk stratification gene signatures [26,27], and early-stage trials are investigating anti-IL2RA therapy for adult AML [26]. Our findings associate IL2RA overexpression with poor EFS in both adult and pediatric AML, suggesting insights from adult trials may be informative to pediatric cases.

The MYH15 (Myosin Heavy Chain 15) gene, primarily involved in muscle physiology [28], has emerging implications in cancer biology. As part of the myosin family, MYH15 contributes to cytoskeletal dynamics, influencing cell motility, shape, and migration [29]. Cytoskeletal alterations are a hallmark of cancer, particularly in metastasis, and MYH15 dysregulation may facilitate AML progression. We observed aberrant MYH15 expression in both adult and pediatric AML, with upregulation linked to poor EFS, consistent with previous studies [30]. Further research is needed to understand its clinical significance.

The SIX3 gene, a homeobox transcription factor, is essential for embryonic development. Recent studies suggest SIX3 and other SIX family genes regulate the proliferation and differentiation pathways implicated in cancer [31]. While its role in AML is unclear, our findings indicate SIX3 overexpression is associated with improved EFS in both adult and pediatric AML, suggesting its potential as a prognostic biomarker [32]. Additional studies are required to confirm this association.

The SOBP (Sine Oculis Binding Protein) gene encodes a transcriptional regulator involved in brain and ear development. It interacts with transcription factors and signaling molecules to influence chromatin remodeling, potentially affecting myeloid differentiation [33,34]. Our analysis links SOBP overexpression to worse EFS in AML, suggesting its role as a biomarker for AML subtypes with dysregulated transcriptional machinery.

Although these five genes are involved in distinct biological processes, their roles in inflammation, immune regulation, cytoskeletal dynamics, transcriptional control, and differentiation suggest potential functional interactions in leukemia biology. For example, F2RL3 and IL2RA are both involved in inflammation and immune regulation, suggesting they may work together to shape the leukemia microenvironment, potentially contributing to immune suppression and tumor progression. Similarly, SIX3 and SOBP regulate transcription and development, influencing leukemia cell differentiation. Additionally, pro-inflammatory signaling (F2RL3 and IL2RA) may interact with transcriptional regulators (SOBP and SIX3) to drive leukemic cell proliferation or hinder normal differentiation, further contributing to AML progression.

Limitations: This study relies on the bioinformatics analyses of diverse gene expression datasets, including RNA-seq and microarray. EFS information was not available for all datasets, and findings were validated only in datasets with accessible clinical information. Additionally, to compare with categorical risk groups based on cytogenetic classification, we discretized the continuous risk score into three approximately equal-sized groups. However, the cutoff points were not optimized, potentially reducing statistical power. The five-gene risk score could yield better performance with optimal cutoff selection [35]. Moreover, when comparing two continuous risk scores from Cox regression, Restricted Mean Survival Time (RMST) provides a more efficient alternative statistical measure for prognostic biomarker comparison [36,37]. RMST curves summarize total survival time across different time horizons, offering a robust summary measure that allows the direct comparison of continuous risk scores without the need for discretization. Finally, in this study, we focused solely on identifying genomic signatures associated with EFS. Other potential factors influencing EFS were not explored.

Future directions: While current cytogenetic and molecular classifications provide valuable prognostic information, our findings suggest that gene expression-based risk scores may offer additional stratification, particularly for patients lacking well-defined genetic markers. As a next step, we plan to investigate the mechanisms linking this signature to AML outcomes, including its interactions with known AML pathways and potential associations with specific disease subtypes or treatment responses. Additionally, we aim to validate our findings in independent cohorts and assess the clinical applicability of the risk score in combination with existing stratification models.

## 5. Conclusions

Existing gene signatures in the literature are predominantly tailored for OS and may not consistently perform optimally in the stratification of EFS risk. Our bioinformatics data analysis has led to the identification of a five-gene signature specifically for EFS in both adult and pediatric AML. The genes comprising this signature are F2RL3, IL2RA, MYH15, SIX3, and SOBP. Importantly, genes regulating immune responses, *F2RL3* and *IL2RA*, are among those that define both pediatric and adult EFS in AML. The role of these genes in the outcomes of immunotherapies in AML patients should be further investigated. Notably, this five-gene signature demonstrates robust performance in both EFS and OS risk stratification when applied to independent datasets. Pediatric AML is a rare but highly aggressive cancer that presents unique challenges in diagnosis, treatment, and research. By identifying shared gene signatures and molecular pathways, we may better understand the disease’s biology, develop targeted therapies, and potentially shorten the time needed to translate adult AML discoveries to pediatric treatments.

## Data Availability

All gene expression data are publicly available and can be downloaded from the following site https://www.cbioportal.org/datasets.

## Supplementary Materials

The following supporting information can be downloaded at: https://www.mdpi.com/article/10.3390/diagnostics15111421/s1, Genes associated with EFS shared between adult and pediatric AML are in the Supplementary Table S1.
